# The SPOP-ITCH Signaling Axis Protects Against Prostate Cancer Metastasis

**DOI:** 10.3389/fonc.2021.658230

**Published:** 2021-07-12

**Authors:** Jinlu Ma, Mengjiao Cai, Yaqi Mo, Joshua S. Fried, Xinyue Tan, Yuan Ma, Jie Chen, Suxia Han, Bo Xu

**Affiliations:** ^1^ Department of Radiation Oncology, The First Affiliated Hospital, Xi’an Jiaotong University, Xi’an, China; ^2^ Department of Oncology, Southern Research Institute, and University Alabama at Birmingham, Birmingham, AL, United States; ^3^ Department of Biochemistry and Molecular Biology, National Clinical Research Center for Cancer, Key Laboratory of Cancer Prevention and Therapy, Tianjin Medical University Cancer Institute and Hospital, Tianjin, China; ^4^ Center for Intelligent Oncology, Chongqing University Cancer Hospital and Chongqing University School of Medicine, Chongqing, China

**Keywords:** SPOP, ubiquitylation, prostate cancer, ITCH, metastasis

## Abstract

Prostate cancer is one of the most common causes of cancer incidence and death in men, with the mortality caused primarily by the late-stage and metastatic forms of the disease. The mechanisms and molecular markers for prostate cancer metastasis are not fully understood. Speckle type Poz Protein (SPOP) is an E3 ubiquitin ligase adaptor that is often mutated in prostate cancer. In this study, we sequenced the *SPOP* gene in 198 prostate cancer patients and found 16 mutations in the cohort. Multivariate analysis revealed that *SPOP* mutations correlated with the clinical stage of the disease and strongly with metastasis. We identified ITCH as a candidate protein for SPOP-mediated degradation *via* mass spectrometry. We demonstrated the interaction between SPOP and ITCH, and found that the *SPOP* F133L mutation disrupted the SPOP-ITCH interaction, leading to a subsequent increase in the ITCH protein level. Further, we found that the SPOP knockdown led to higher levels of Epithelial- mesenchymal transition (EMT) proteins and increased cell invasion. Together, our results highlight the functional significance of the SPOP-ITCH pathway in prostate cancer metastasis.

## Introduction

Prostate cancer is among the leading causes of both cancer incidence and death in men (1). The mortality from prostate cancer is largely due to advanced late-stage and metastasis (2). There is a pressing need to elucidate the underlying molecular drivers that render patients susceptible to the more aggressive forms of this disease, in order to develop effective therapies. Previous genetic studies have found typical mutations/aberrations in prostate cancer, including ERG gene fusions, p53 aberrations, Androgen Receptor (AR) amplifications, and Speckle type Poz Protein (SPOP) missense mutations (3). SPOP is an E3 ubiquitin ligase adaptor that forms a complex with Cullin 3 to mediate ubiquitylation and destruction of target proteins (4, 5). Recent studies on prostate cancer patients have revealed that the *SPOP* gene as the most common recurrent point mutations, with 8%–15% of the patient population carrying a somatic mutation in this gene (6–11). Of these naturally occurring *SPOP* mutations, a significant proportion occurs in the substrate-binding MATH domain. Screening studies, along with the Cancer Genome Atlas (TCGA) database, have demonstrated that *SPOP* mutations represent an early event during disease development, and they are largely exclusive with the common ETS gene fusion events in prostate cancer, making *SPOP*-mutant tumors a distinct subgroup of prostate cancer (12). Many substrates of SPOP are known oncoproteins, such as SRC3, AR, and ERG. Owing to its role in regulating drivers of oncogenesis, SPOP has emerged as a potent tumor suppressor in prostate cancer. However, little is known about the prognostic value of the *SPOP* mutational status, and in-depth studies are needed to better understand the clinical impact of *SPOP* mutations.

ITCH (Itchy Homolog) is also an E3 ubiquitin ligase, and ITCH-mediated ubiquitylation is essential in multiple cellular functions, including the immune response, hematopoiesis, and lipid turnover (13–15). In the context of cancer, ITCH can be both anti- or pro-tumorigenic. On one hand, studies have shown ITCH acting as a tumor suppressor by regulating HER2 and FLIP in breast and brain cancers, respectively (16–20). On the other hand, evidence has suggested that ITCH might also function as a tumor promoter by downregulating Smad7, prompting TGF-β to promote Epithelial Mesenchymal Transition (EMT) in breast cancer cells (21, 22). These observations suggest that the role of ITCH in oncogenesis is subtype- and/or context-dependent.

In the present study, we sequenced the *SPOP* gene in a cohort of 198 prostate cancer patients in China. The multivariate analysis identified SPOP mutations as an independent predictor of metastasis in the patients. We then carried out a proteomic analysis to identify substrates that could be involved in metastasis, and we identified ITCH as the primary target for SPOP. We demonstrate that the SPOP-ITCH signaling pathway plays a critical role in prevention of prostate cancer metastasis.

## Methods

### Patient Information

198 primary prostate tumor patients (stage I-IV) diagnosed at the Cancer Center in the First Affiliated Hospital of Xi’an Jiaotong University, from January 2010 to December 2015 were enrolled in the study. Among these patients, the age at diagnosis ranged from 44 to 91 years, with a median age of 70 years. In all cases, the staging evaluation involved the recording of medical history and a physical examination, including a digital rectal examination, serum PSA (Prostate Serum Antigen), computed tomographic (CT) scan of the pelvis or pelvic coil magnetic resonance imaging (MRI) scan of the prostate and pelvis, bone scan, and a transrectal ultrasound-guided needle biopsy of the prostate with Gleason score histologic grading. The clinical stage of the disease was identified from the findings of the digital rectal examination, in accordance with the 2002 American Joint Committee on Cancer (AJCC) staging system. Tumor size was defined as the maximum tumor diameter measured by pelvic coil MRI scan at the time of diagnosis. Follow-up data were available for all 198 patients, and the median length of follow-up was 27 months (range: 5 to 70 months). The study was approved by the Research and Ethical Committee of the First Affiliated Hospital of Xi’an Jiaotong University.

### SPOP Mutation Analysis

DNA was extracted from frozen cancer tissues collected from the patients. A 25–30 mg mass of tissue was homogenized from each sample, followed by DNA extraction from the homogenate and its quantification using the Picogreen dsDNA Quantitation Reagent (Invitrogen, Carlsbad, CA, USA). Mutational analysis of *SPOP* was performed using a set of four primer pairs that covered the coding region of exons 6 and 7 of the *SPOP* gene. All fragments were sequenced with the BigDye Terminator Cycle Sequencing Kit and ABI 3730 automated sequencer (Applied Biosystems, Foster City, CA, USA). Each mutation was confirmed in duplicate samples.

### Cell Culture

Human prostate cancer cell lines LN-CaP and PC-3 (from American Type Culture Collection) were cultured in DMEM (GIBCO-Invitrogen, Carlsbad, CA) containing 10% FBS, 2 mM L-glutamine, 100 units/mL of penicillin, and 100 mg/mL streptomycin, and grown under standard cell culture conditions at 37°C in a humidified atmosphere with 5% CO_2_. Human embryonic kidney HEK293T cells (American Type Culture Collection) were cultured in DMEM high-glucose (Invitrogen) with 10% (vol/vol) FBS (Invitrogen) in a 5% (vol/vol) CO_2_ incubator at 37°C.

### siRNA Depletion of SPOP

For RNAi experiments, ON-TARGET plus double-stranded siRNA oligomers against human SPOP and non-specific scrambled siRNA control (Stealth RNAiTM siRNA Negative Control, Med GC) were purchased from Thermo Scientific. Cells were transfected with Lipofectamine RNAiMAX (Invitrogen) with a final siRNA concentration of 50 nM, according to the manufacturer’s instructions.

### Expression Constructs

Using Phusion High-Fidelity DNA Polymerase (New England BioLabs, Ipswich, MA, USA) and PCR primers containing a Flag tag at the N terminus, expression constructs of Flag-tagged *SPOP* (pcDNA3.1-Flag-*SPOP*, wild-type) was generated by insertion of the PCR-amplified *SPOP* cDNA-coding sequence into mammalian expression vector pcDNA3.1 Hygro (+) (Invitrogen). *In vitro* site-directed mutagenesis was used to obtain the Flag-tagged SPOP mutants by two PCR amplifications, using pcDNA3.1-Flag-*SPOP* as the template. PCR-amplified DNA strands coding for the *SPOP* mutants were inserted into pcDNA3.1 Hygro (+) (Invitrogen) to generate the corresponding mammalian expression vectors: pcDNA3.1-Flag-*SPOP*-F133L.

### Mass Spectrometry (MS)

Cells were lysed in buffer [20 mM Tris–HCl, pH 7.3, 300 mM KCl, 0.2 mM EDTA, 0.5% Triton, 10% glycerol, 1 mM phenylmethylsulfonyl fluoride, 10 mM glycerophosphate, and 0.1 mM Na_3_VO_4_, and protease inhibitors]. Total protein (50 mg) was IP with anti-SPOP (1:1000; Abcam) or unspecific mouse monoclonal IgG antibodies (I-1000; GeneTex Inc.) and 50 μl protein G-conjugated beads (Thermo Fisher). Washed beads were separated by SDS-PAGE. Following Coomassie Blue Staining, protein bands were excised and digested. After separation on a reversed phase LC column, eluted peptides were analyzed on a MALDI-QIT-TOF-based mass spectrometer with electrospray ionization (Micromass/Waters). The MS/MS data were processed using Masslynx software (Micromass), and the MASCOT (Matrix Science) search engine was used to search the NCBI non-redundant database. Protein identifications were based on a minimum random probability score of 25 and with a mass accuracy of 0.1 Da.

### Western Blot

Cells in 6-well tissue culture plates were lysed in 0.5 mL of lysis buffer (PBS containing 1% Triton X-100 and 1 mM PMSF) at 4°C for 10 min. Equal quantities of protein were subjected to SDS–PAGE under reducing conditions. Following a transfer to immobilon-P transfer membrane, successive incubations with a primary antibody and a horseradish peroxidase-conjugated secondary antibody were carried out for 60–120 min at room temperature. The immunoreactive proteins were then detected using the ECL system. Films showing immunoreactive bands were scanned using an HP Scanjet 5590 scanner (HP, Pal Alto, CA, USA). The antibodies used for Western blotting were: mouse anti-HA (Roche, Basel, Switzerland), monoclonal mouse anti-FLAG M2 (Sigma, St. Louis, Mo, USA), anti-SPOP (Abcam, Cambridge, United Kingdom), monoclonal rabbit anti–ITCH (Cell Signaling, Danvers, MA, USA), rabbit anti-E-cadherin (Santa Cruz, Dallas, TX, USA), mouse anti-FLAG-HRP (Sigma), mouse anti-HA-HRP (Roche), anti-rabbit IgG-HRP (Sigma), and anti-mouse IgG-HRP (Sigma).

### Co-Immunoprecipitation

For co-immunoprecipitation analysis of Flag-SPOPs with ITCH, cell lysates containing an approximately equal amount of each expressed Flag-SPOP (WT or mutant) and ITCH protein were combined. The Flag-SPOP/ITCH cell lysate mix was incubated overnight with anti-Flag M2 affinity beads (Sigma) at 4°C with constant rotation, before protein A Dynabeads (Invitrogen) were added to collect the immune-complex. The beads were washed four times with lysis buffer and boiled in Laemmli sample-loading buffer for 10 min to elute the precipitated proteins. The supernatants were separated by SDS/PAGE, and immunoblotting was used to detect Flag-SPOPs and ITCH. The input was loaded at 1/10th of the total lysate amount subjected to each immunoprecipitation experiment.

### Ubiquitylation Assay

HEK293 cells were transfected with indicated siRNA or control, and treated with MG132 (10 μM) for 8 h before harvest. The harvested cells were lysed with NETN buffer containing protease inhibitors (Roche). After sonication, the supernatants were incubated with anti-Flag M2 affinity beads for 4–6 h at 4°C. The beads were then spun down, washed with the NETN lysis buffer, and prepared for western blot analysis.

### Migration and Invasion Assay

Approximately 5 × 10^4^ cells transfected for 48 h with siRNA were reseeded in the upper chamber of transwell assay inserts to test for migration. Cells suspended in serum-free medium were added to the upper chamber, and medium with 10% FBS was added into the lower chamber. After 24 h, nonmigratory cells on the upper side of the inserts were carefully removed. Migratory cells on the undersurface of the inserts were fixed with methyl alcohol, and stained with crystal violet. To test invasion, the upper chamber was precoated with matrigel. The subsequent steps were the same as those in the migration assay.

### Scratch Wound Healing Assay

Treated cells were suspended in 6-well tissue culture plates. After 24 h, a linear wound was scratched on the confluent monolayer of cells using a sterile 20-μL pipette tip, and the medium was immediately changed with a fresh one. Cells were imaged at 0, 24, and 48 h after scratch.

### Statistical Analysis

Fisher’s exact tests were used to compare categorical data. The risk of metastasis among patients with an *SPOP* mutation was evaluated using univariate and multivariate Cox regression analysis. Cox proportional hazards regression models were used to test the prognostic role of the *SPOP* mutation status [Hazard ratios (HR) and 95% confidence intervals (CI)]. Two-sided P-values lower than 0.05 were considered statistically significant. All analyses were performed using the IBM SPSS Statistics for Windows, Version 20.0. (Armonk, NY, USA).

## Results

### Patient Characteristics and the Frequency of SPOP Mutations

Listed in **Table 1** are patient characteristics in this study. According to the AJCC staging system standards, 99 cases were stage I-II, 32 were stage III, and 67 were stage IV. 84.9% of the patients had localized disease, whereas the other 15.1% had metastasis at the time of diagnosis. We screened for somatic variants in Exons Six and Seven of the *SPOP* gene in the prostate tumor tissues, as all the previously reported *SPOP* mutations were within these two exons. Three somatic *SPOP* missense mutations were identified in 16 out of the 198 tumor tissues (8%). The patients with mutant *SPOP* have mutations in the following codons (**Supplementary Table 1**): phenylalanine (F) to valine (V), leucine (L), or cysteine (C) substitution in codon 133 (F133V/L/C) (n = 7), tryptophan (W) to glycine (G), cysteine (C), or serine (S) substitution in codon 131 (W131G/C/S) (n = 6), tyrosine (Y) to cysteine (C) or serine (S) substitution in codon 87 (Y87C/S) (n = 2), and phenylalanine (F) to leucine (L) substitution (n = 1). As shown in **Table 1**, the mutation frequency of *SPOP* was associated with the T stage and Risk Group. However, it did not correlate with the age of patient, prior treatment PSA level, or the Gleason score. Interestingly, the occurrence of *SPOP* mutations was significantly associated with metastasis (P=0.00001). Among patients with *SPOP* mutations, 56.3% showed metastasis at the time of diagnosis of primary cancer, compared with only 11.5% of the patients with wild-type *SPOP* (OR, 6.58, 95% CI, 5.81-7.46, P = 0.000) (**Table 2**). The metastasis risk for patients with SPOP mutations was thus 1.27 times higher than that of the SPOP wild-type patients (OR, 1.27, 95% CI, 1.08-1.48, P = 0.003).

**Table 1:** Clinical characteristics of 198 patients with prostate cancer included in the study.

Clinic Characteristic	Cases (N=198)	SPOP Mutation		*p*-Value
		Yes (N)	No (N)	
**Age**				0.82
≥75	61	6	55	
65-74	83	7	76	
55-64	45	3	42	
≤54	9	0	9	
**Prior treatment PSA level**				0.32
≤10	36	2	34	
10.1 - 20	39	3	36	
20.1 - 40	45	3	42	
≥40	78	8	70	
**Gleason Score**				0.17
≤6	50	3	47	
3+4/4+3	109	7	102	
≥8	39	6	33	
**Stage**				**0.006**
I-II	99	1	95	
III	32	5	25	
IV	67	10	61	
**PSA Recurrence**				**0.008**
Yes	26	6	20	
No	172	10	162	
**Risk Group**				**0.02**
Low	36	0	35	
Intermediate	108	5	102	
High	54	11	45	
**Metastatic**				**0.00001**
Yes	30	9	21	
No	168	7	161	

Bold values mean the p-values < 0.05.

**Table 2:** Associations between metastatic reporting at the first diagnosis and SPOP mutational statuses.

SPOP	Metastatic prostate cancer No. of Men (%)		Univariate analysis		Multivariate analysis	
	Yes (N=30)	No (N=168)	OR (95% Cl)	*p*-Value	OR (95% Cl)	*p*-Value
Mutated (N=16)	9 (56.3)	7 (43.8)	6.58 (5.81-7.46)	**0.000**	1.27 (1.08-1.48)	**0.003**
Wild-Type (N=182)	21 (11.5)	161 (88.5)				

Bold values mean the p-values < 0.05.

### Identification of ITCH as a Substrate of SPOP

As the analysis revealed that *SPOP* mutations correlated strongly with metastasis, we sought to uncover a potential mechanism for this phenomenon. We used siRNA to suppress the SPOP expression in prostate cancer cells, and performed a mass spectrometry analysis on the lysates from these cells to determine which proteins have an altered expression upon the depletion of SPOP. Potential candidate proteins were selected based on increased protein levels when SPOP was knocked down, a previously published role in oncogenesis, and the presence of a SPOP degron. The SPOP degron we used was Φ-Π-S-S/T-S/T (Φ-nonpolar; Π-polar), based on the findings in Zhuang M et al. (5). We then classified the proteins based on whether they showed an increased or decreased expression after *SPOP* knockdown. There were 15 proteins on the list (**Supplementary Table 2**). Focusing on the proteins with an increased expression, we hypothesized that these proteins could be potential SPOP substrates for ubiquitylation-mediated degradation. Within this subset of proteins, we then focused on ITCH for validation and further experimentation, as evidence suggests that it contributes to EMT.

**Figure 1:**
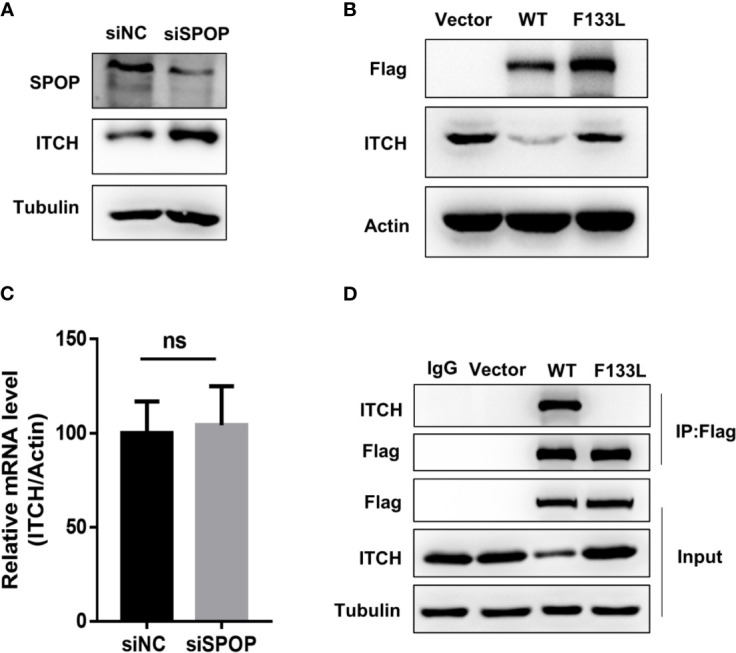
SPOP interacts and downregulates ITCH. **(A)** Protein expression of lysates derived from LN-CaP cells transfected with either a control or SPOP -siRNA, measured by Western blot. **(B)** Protein expression of lysates derived from LN-CaP cells transfected with an empty vector, the wild type or F133L mutant form of SPOP, measured by Western blot. **(C)** ITCH’s mRNA expression in the transfected cells, measured by RT-PCR. **(D)** Interaction of SPOP and ITCH was assessed by co-immunoprecipitation of lysates derived from PC-3 cells expressing empty vector, the wild type or F133L mutant SPOP using IgG or the anti-Flag antibody. Membranes were probed with the indicated antibodies. ns, no significance.

We began our validation by using siRNA to knockdown SPOP expression in prostate cancer cells followed by measuring the expression of ITCH by Western blot. Knockdown of SPOP caused a dramatic increase in ITCH expression, which was not observed in cells treated with the control siRNA (**Figure 1A**). We then tested if the clinically relevant *SPOP* mutations also regulate the ITCH expression. We focused on the F133L mutation, which represented one of the most frequent mutants in the clinical cohort. Expression of the wild-type SPOP caused a decrease in ITCH expression compared with the F133L mutation and vector-transfected cells (**Figure 1B**). However, the mRNA level was not affected (**Figure 1C**). From these data, we conclude that SPOP regulates the turnover of the ITCH protein. We then performed co-immunoprecipitation to determine whether SPOP and ITCH interact with each other. We transfected PC-3 cells with plasmids containing the wild-type or F133L mutant form of *SPOP*. As expected, wild-type SPOP could interact with ITCH, but the F133L mutation abrogated the SPOP-ITCH interaction (**Figure 1D**). Taken together, these results suggest that ITCH interacts with SPOP, and that this interaction is abolished by the F133L mutation.

### SPOP Is Essential for ITCH Ubiquitylation

**Figure 2:**
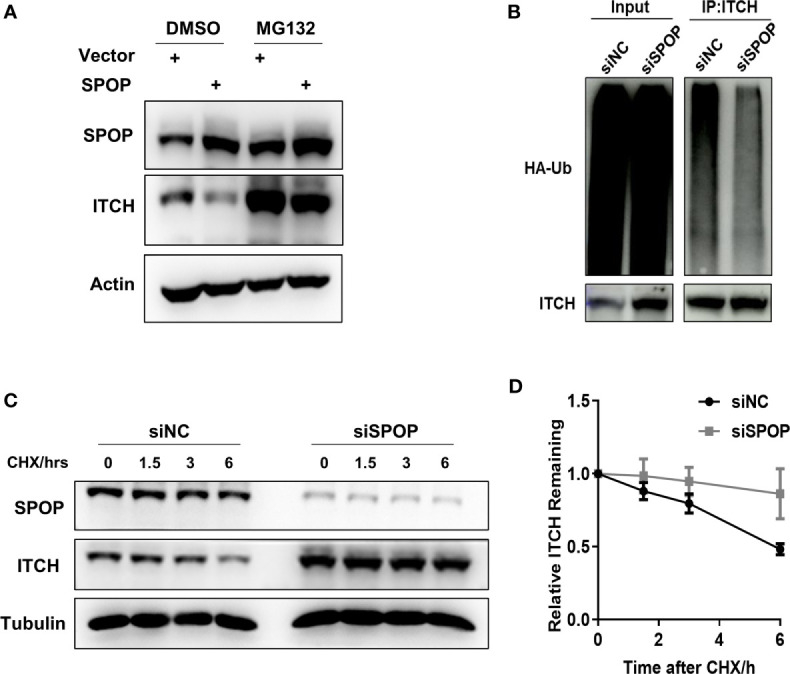
SPOP is required for ITCH ubiquitylation and it suppresses ITCH stability. **(A)** LN-CaP cells transfected with either vector or HA-tagged SPOP were treated MG132. Protein levels were detected by Western blot using indicated antibodies. **(B)** The ubiquitylation assay of ITCH in the presence or absence of SPOP. HEK293 cells were co-transfected with HA-ubiquitin and the control or SPOP siRNAs. Endogenous ITCH was immunoprecipitated and immunoblotted with the anti HA or ITCH antibody**. (C)** SPOP knockdown increased the half-life of ITCH protein. CHX, cycloheximide. **(D)** Quantification of three independent experiments performed in C.

Next, we investigated the biochemical significance of the SPOP-ITCH interaction. If the SPOP-ITCH interaction is to regulate the ITCH protein stability by increasing its proteasomal degradation, then the proteasome inhibitor MG132 should reverse the level of ITCH in SPOP-overexpressed cells. Indeed, the upregulation of ITCH by SPOP overexpression was reversed by MG132 (**Figure 2A**). To further understand the regulation of ITCH by SPOP, we performed a denaturing lysis on lysates from HEK293 cells co-transfected with HA-ubiquitin, and a control siRNA or a SPOP-targeting siRNA. Endogenous ITCH was immunoprecipitated and blotted with the anti HA-ubiquitin antibody. We observed that SPOP knockdown reduced ubiquitylation signals (**Figure 2B**). We also measured the protein stability of ITCH in LN-CaP cells, and found that ITCH was more stable in SPOP siRNA-transfected cells than in control siRNA-transfected cells, as revealed by a cycloheximide time course experiment (**Figures 2C, D**). From the above findings, we conclude that SPOP may contribute to the protein stability of ITCH through ubiquitylation.

### SPOP Depletion Promotes EMT and Enhances Invasion and Migration in Prostate Cancer Cells

**Figure 3:**
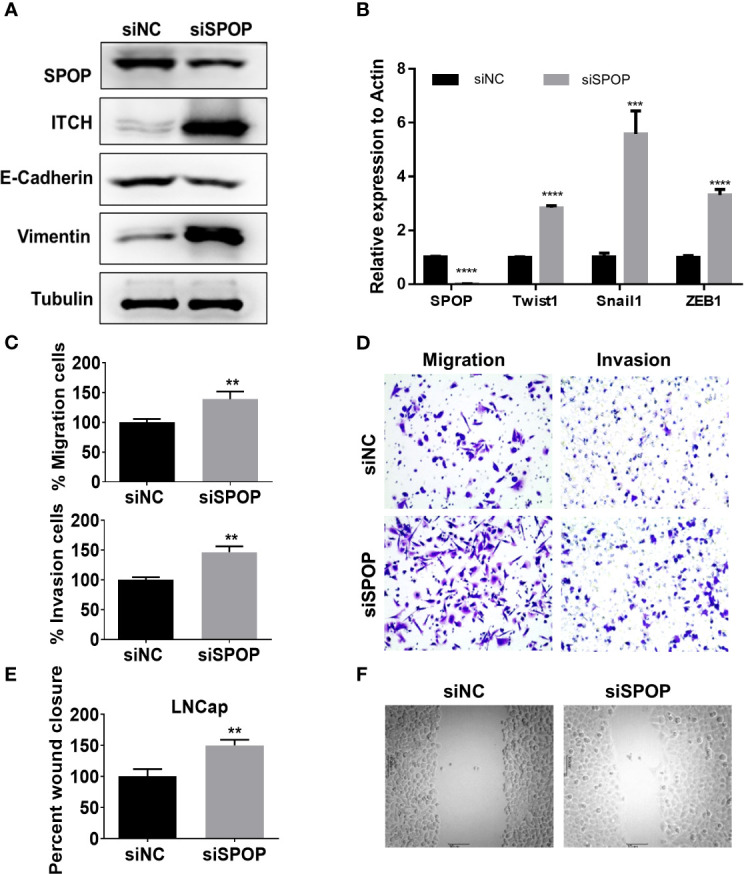
Downregulation of SPOP promotes EMT. LN-CaP cells were transfected with control or SPOP siRNA, before they were subjected for the following assays: **(A)** Protein expression of E-Cadherin and Vimentin measured by Western blotting. **(B)** mRNA expression for TWIST, SNAIL and ZEB1measured by RT-PCR analyses. **(C, D)** Cell migration and invasion measured by the Modified Boyden chamber assay. **(E, F)** Cell migration measured by the wound-healing migration assay. **P < 0.01;***P < 0.001; ****P < 0.0001.

To examine the connection between the SPOP-ITCH interaction and metastasis, we probed if E-Cadherin, an inhibitor of EMT (23–25), was downregulated in SPOP knockdown cells. We began by investigating if SPOP knockdown and the subsequent ITCH increase changed the E-cadherin protein level. We found that *SPOP* knockdown cells showed a marked reduction in the E-cadherin level, with a concomitant rise in the ITCH protein level (**Figure 3A**). Meanwhile, vimentin expression in LN-CaP cells increased after *SPOP* knockdown. We also assessed the mRNA level of EMT markers, including Snail1, Twist1, and ZEB1, after the silencing of *SPOP*. We found that, in the absence of SPOP, LN-CaP cells increased the transcriptional expression of these markers (**Figure 3B**). We also conducted transwell and scratch wound healing assays to test the effect of SPOP-depletion on prostate cell invasion and migration. We found that *SPOP* knockdown significantly promoted LN-CaP cell migration and invasion (**Figures 3C–F**). Collectively, we demonstrate that SPOP knockdown is associated with a more mesenchymal phenotype in prostate cancer cells.

### SPOP Mutations Are Associated With Biochemical Failure

**Figure 4:**
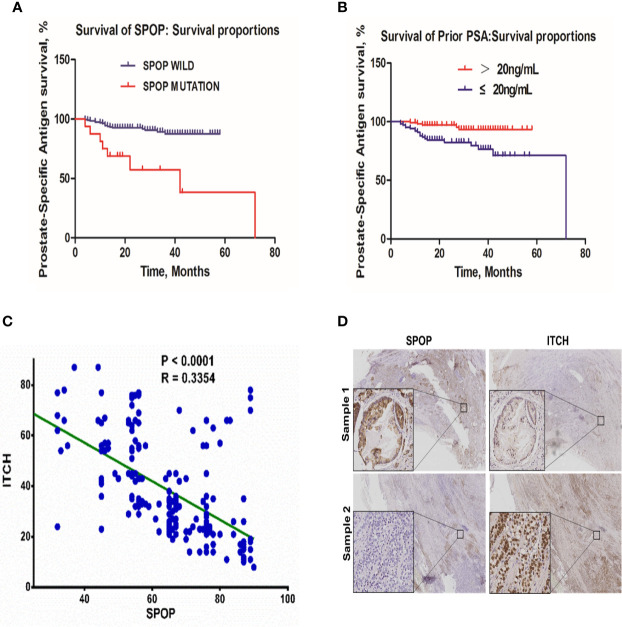
The biochemical failure in the patient cohort. **(A)** Association of biochemical failure with SPOP mutations; **(B)** biochemical failure with prior PSA levels; **(C)** Linear regression correlation analysis of SPOP and ITCH expression levels. **(D)** Representative images of immuno-histochemical staining of SPOP and ITCH in prostate cancer tissue samples.

Among the 198 patients, 26 showed evidence of biochemical relapse. The actuarial biochemical failure with SPOP mutation was 37.5% and 11.0% for patients with mutant and wild-type SPOP, respectively (P < 0.0001) (**Figure 4A**). Meanwhile, the correlation of the pre-treatment PSA level with the biochemical failure was 6.7% and 17.1% for patients with a PSA level of ≤ 20 and >20 ng/mL, respectively (P = 0.0021) (**Figure 4B**). However, there was no significant difference between the age groups of ≤65 and >65 year-old-patients (P = 0.527) (**Supplemental Figure 2**).

### Higher SPOP Expression Is Associated With Lower ITCH Expression in Clinical Samples

To validate the *in vitro* data, we analyzed the protein expression levels of SPOP and ITCH in these samples *via* immunohistochemistry staining. We found that a higher expression of SPOP was associated with lower ITCH expression. The linear regression analysis on the immunohistochemistry data indicated a negative correlation between them (R=0.33) (**Figure 4C**, and representative images are shown in **Figure 4D**), further supporting the conclusion derived from the *in vitro* studies.

## Discussion

Recent studies have indicated that somatic mutations in the *SPOP* gene are cancer-driven and might affect the therapeutic response. In this study, we began by analyzing the clinical characteristics of a patient cohort in China and their association with *SPOP* mutations. Despite the well-known SPOP mutational spectrum in Western countries, it is still less clear about SPOP mutation in Chinese patients. Our study represents the largest cohort for prostate cancer thus far. The mutation rate is about 8%, which is consistent with the TCGA data. Interestingly, a multivariate analysis revealed the presence of *SPOP* mutations as an independent predictor of prostate cancer metastasis. Based on the proteomic analysis and *in vitro* validation, we identified ITCH as a novel SPOP substrate. We find that SPOP is involved in ubiquitin-mediated ITCH degradation. Mutation in *SPOP* interrupts the interaction and subsequently led to an increase on the ITCH level, which results in elevated levels of EMT-associated proteins and enhanced cellular invasiveness. Together our data highlight the role of the SPOP-ITCH axis in protection against prostate cancer metastasis. Our observations are supported by previous reports, which showed that SPOP mutations increased the motility of prostate cancer cell *in vitro* and *in vivo* (26, 27). There are some caveats to our study. First, our patient cohort was relatively small (198 cases). Larger and more diverse patient groups are needed to validate the prognostic value of *SPOP* mutations. Confirming the relationship between mutated SPOP and ITCH in patients also require a larger number of patient cohort. Second, while our analysis did find a strong correlation between *SPOP* mutations and metastasis, other biomarkers were not considered. Combinations with other known biomarkers are likely to provide better prognostic values for predicting metastasis. Third, although we have outlined a molecular mechanism that considers the disruption of the SPOP-ITCH pathway as a potential driver of metastasis, we cannot rule out the contribution of other SPOP substrates. Further investigations are needed to determine the contribution of additional SPOP substrates in disease progression. Despite the fact that a couple of previous studies have concluded that the SPOP mutation status has little-to-no predictive value for the course of the disease (9, 28), there is evidence that SPOP mutations does have a poor prognosis based on the expression of SPOP. This is in contrast to the conclusions we obtained in the current study. A potential reason for this discrepancy is that the patient cohort enrolled in this study has the disease in more advanced stages. Moreover, the above mentioned studies were conducted largely in men of Caucasian or African descent, whereas every patient in our study was of Chinese descent. Biologically, there are also other limitations in the study. For example, whether SPOP mutation associated EMT is ITCH-dependent remains to be proven in additional cell models. In addition, the direct interaction between SPOP and ITCH needs to be further established, due to the fact that a secondary interaction of SPOP and ITCH might be mediated by a different protein.

Owing to a relatively high frequency of *SPOP* somatic mutations in prostate cancer and the deadly nature of metastasis, our results have a clear clinical implication. In an era of medicine where tumors are being sequenced frequently and that personalized medicine is becoming increasingly common, our observations have potential to guide treatment strategies. Patients with mutated *SPOP* might have a significantly higher risk for developing advanced stages of the disease compared with others that have a wild-type SPOP, and they may be assigned more aggressive treatment regimens. Our findings could also be potentially expanded to other cancers, wherein *SPOP* is found to be mutated (endometrial and thyroid cancers) or its protein levels reduced (e.g., gastric, colon, and breast cancers).

## Data Availability Statement

The original contributions presented in the study are included in the article/**Supplementary Material**. Further inquiries can be directed to the corresponding author.

## Ethics Statement

The studies involving human participants were reviewed and approved by the Research and Ethical Committee of the First Affiliated Hospital of Xi’an Jiaotong University. The patients/participants provided their written informed consent to participate in this study. Written informed consent was obtained from the individual(s) for the publication of any potentially identifiable images or data included in this article.

## Author Contributions

BX and JLM contributed to the conception of the study. JLM, MC, YMo, JF, XT, YMa, JC, and SH performed the experiments. JLM, MC, and YMo performed the data analyses. JLM, MC, YMo and BX wrote the manuscript. JF, XT, YMa, JC, and SH helped perform the analysis with constructive discussions. All authors contributed to the article and approved the submitted version.

## Funding

This work was supported by grants from the China National Natural Scientific Foundation (81972861, 81672743 and 81472795), and the Shaanxi Province International Cooperation (2018KW-059).

## Conflict of Interest

The authors declare that the research was conducted in the absence of any commercial or financial relationships that could be construed as a potential conflict of interest.
