# Supplementary figures and images for: The SPOP-ITCH Signaling Axis Protects Against Prostate Cancer Metastasis
Source: Front Oncol. 2021 Jul 12;11:658230. doi: 10.3389/fonc.2021.658230 (PMC8311740; doi:10.3389/fonc.2021.658230)

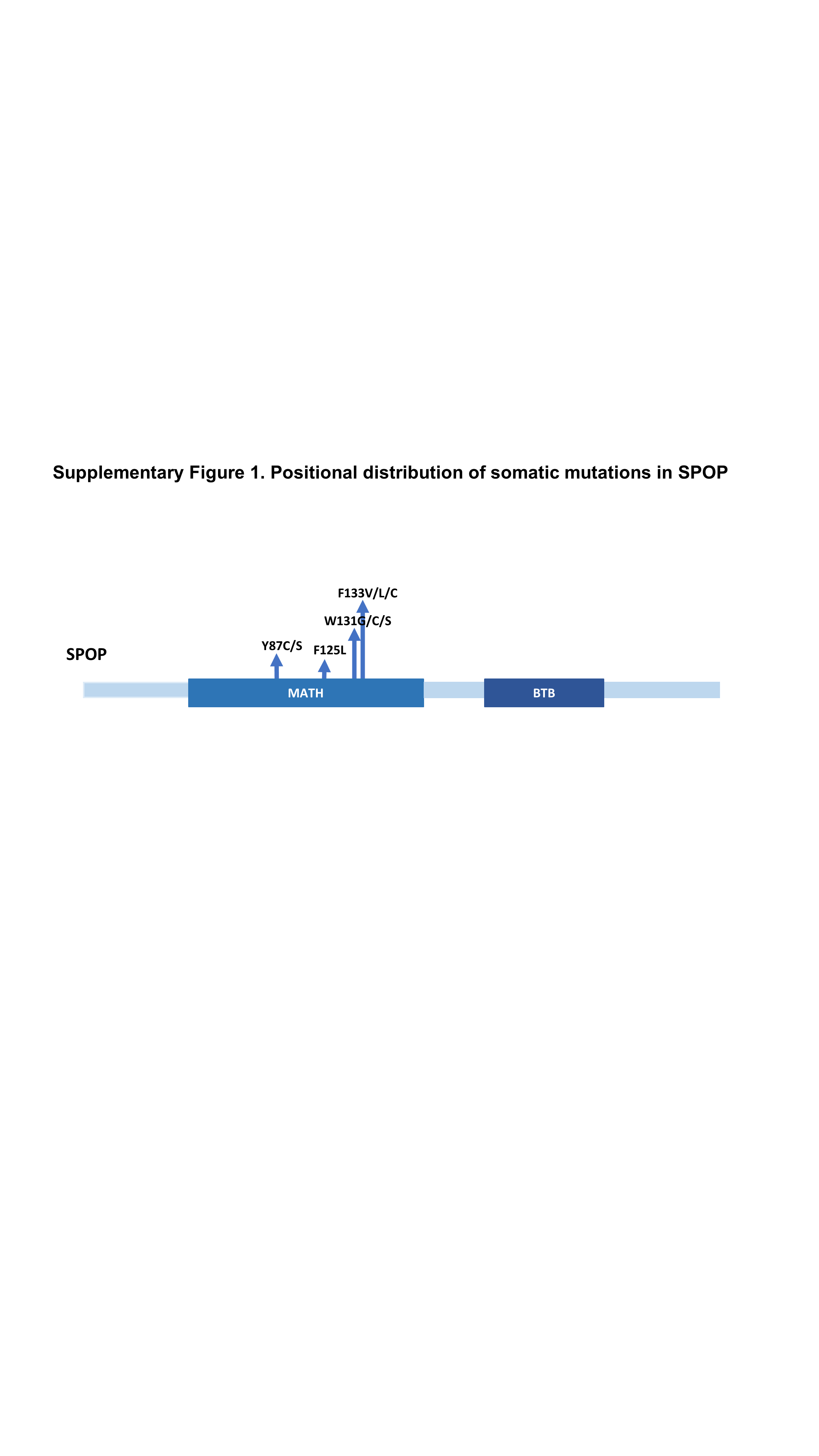

Supplement: Supplementary file 1 [file Image_1.tif]

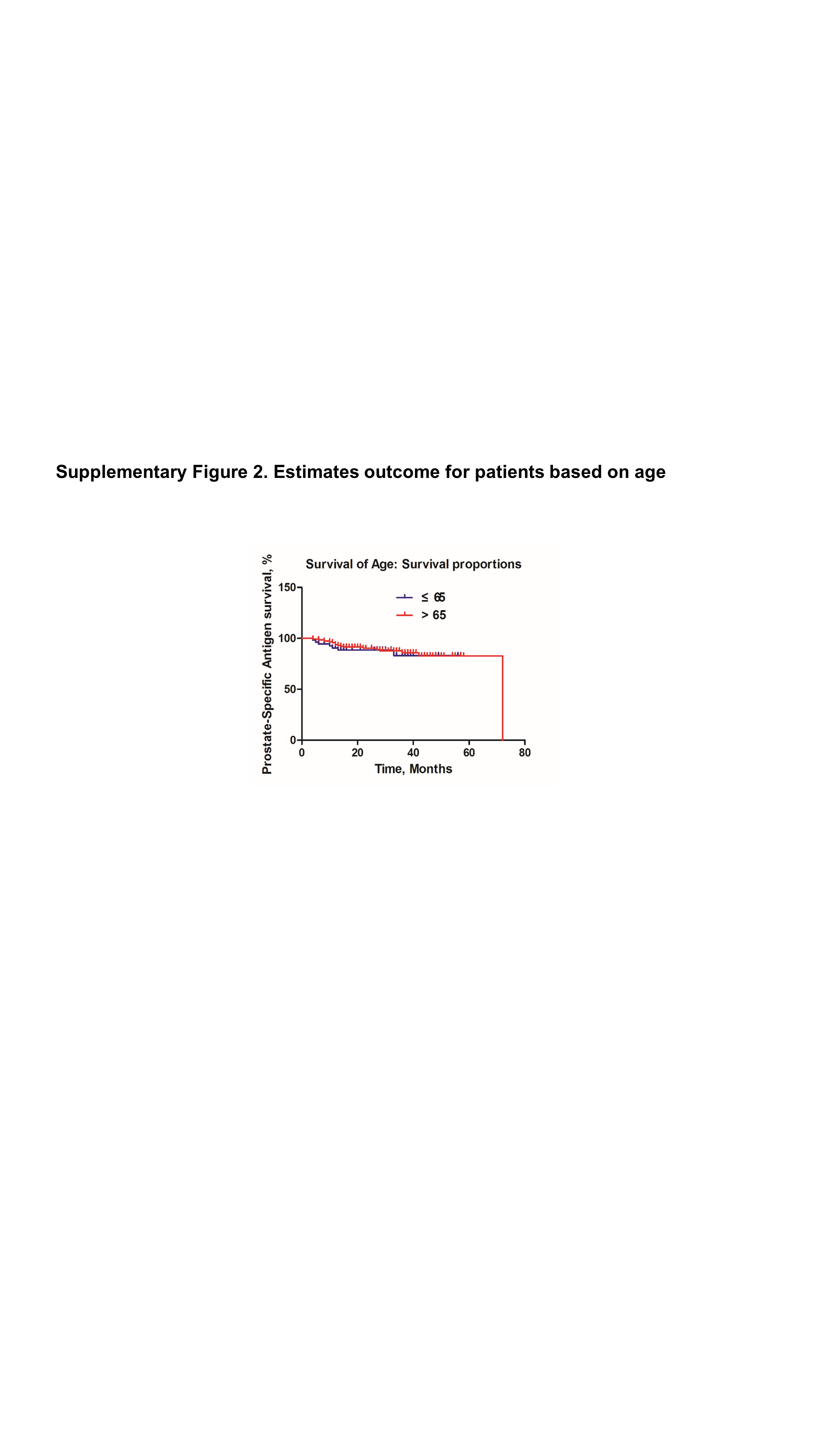

Supplement: Supplementary file 2 [file Image_2.tif]
