# Supplementary material for: The SPOP-ITCH Signaling Axis Protects Against Prostate Cancer Metastasis
Source: Front Oncol. 2021 Jul 12;11:658230. doi: 10.3389/fonc.2021.658230 (PMC8311740; doi:10.3389/fonc.2021.658230)
Supplement: Supplementary file 3 [file Table_1.docx]

**Supplementary Table 1: Localization and frequencies of SPOP mutations**

| **SPOP Mutation (N=16)** | |
| --- | --- |
| **AA Change** | **N (%)** |
| F133V/L/C | 7 (43.8 %) |
| W131G/C/S | 6 (37.5 %) |
| Y87C/S | 2 (12.5 %) |
| F125L | 1 (6.3 %) |
