# Supplementary material for: The SPOP-ITCH Signaling Axis Protects Against Prostate Cancer Metastasis
Source: Front Oncol. 2021 Jul 12;11:658230. doi: 10.3389/fonc.2021.658230 (PMC8311740; doi:10.3389/fonc.2021.658230)
Supplement: Supplementary file 4 [file Table_2.docx]

**Supplementary Table 2: Potential SPOP degradation targets with multiple S/T-rich degron.**

| **Protein Name** | **Has degron?** | **more details** | |
| --- | --- | --- | --- |
| HSPA8 | yes | 1 partial |  |
| PSMA6 | yes |  |  |
| HSPB1 | yes |  |  |
| CPT1A | yes |  |  |
| PRKDC | yes |  |  |
| ITCH | yes |  |  |
| STK26 | yes |  |  |
| TSR3 | yes |  |  |
| H2AFY | yes |  |  |
| HSPA1B | yes | 1 partial, 1 full |  |
| HSPA8 | yes | 1 full |  |
| HSPA8 | yes | 2 full |  |
| HSPA8 | yes | 3 full |  |
| CCT8 | yes |  |  |
| PSMC1 | yes |  |  |
| PRKDC | yes | 1 partial |  |
| PRKDC | yes | 5 partials, 1 full very large protein |  |
| PRKDC | yes | 5 partials, 1 full very large protein |  |
| NUP160 | yes | 5 partials, 1 full very large protein |  |
| PAWR | yes | 2 partial, 1 full |  |
